# PmiRDiscVali: an integrated pipeline for plant microRNA discovery and validation

**DOI:** 10.1186/s12864-019-5478-7

**Published:** 2019-02-13

**Authors:** Dongliang Yu, Ying Wan, Hidetaka Ito, Xiaoxia Ma, Tian Xie, Tingzhang Wang, Chaogang Shao, Yijun Meng

**Affiliations:** 1 College of Life and Environmental Sciences, Hangzhou Normal University, Xuelin Street 16#, Xiasha, Hangzhou, 310036 People’s Republic of China; 2 Faculty of Science, Hokkaido University, Kita10 Nishi8, Kita-ku, Sapporo, Hokkaido 060-0810 Japan; 3 Holistic Integrative Pharmacy Institutes, Hangzhou Normal University, Wenyixi Road 1378#, Hangzhou, 311121 People’s Republic of China; 4 Key Laboratory of microbiological technology and Bioinformatics in Zhejiang Province, Hangzhou, 310036 People’s Republic of China; 5 College of Life Sciences, Huzhou University, Huzhou, 313000 People’s Republic of China

**Keywords:** Plant microRNA, Degradome sequencing (degradome-seq), Secondary structure, Conservation, Processing, Graphic outputs

## Abstract

**Background:**

MicroRNAs (miRNAs) constitute a well-known small RNA (sRNA) species with important regulatory roles. To date, several bioinformatics tools have been developed for large-scale prediction of miRNAs based on high-throughput sequencing data. However, some of these tools become invalid without reference genomes, while some tools cannot supply user-friendly outputs. Besides, most of the current tools focus on the importance of secondary structures and sRNA expression patterns for miRNA prediction, while they do not pay attention to miRNA processing for reliability check.

**Results:**

Here, we reported a pipeline PmiRDiscVali for plant miRNA discovery and partial validation. This pipeline integrated the popular tool miRDeep-P for plant miRNA prediction, making PmiRDiscVali compatible for both reference-based and de novo predictions. To check the prediction reliability, we adopted the concept that the miRNA processing intermediates could be tracked by degradome sequencing (degradome-seq) during the development of PmiRDiscVali. A case study was performed by using the public sequencing data of *Dendrobium officinale*, in order to show the clear and concise presentation of the prediction results.

**Conclusion:**

Summarily, the integrated pipeline PmiRDiscVali, featured with degradome-seq data-based validation and vivid result presentation, should be useful for large-scale identification of plant miRNA candidates.

**Electronic supplementary material:**

The online version of this article (10.1186/s12864-019-5478-7) contains supplementary material, which is available to authorized users.

## Background

As one of the well-known small RNA (sRNA) species, microRNAs (miRNAs) play essential regulatory roles in diverse biological processes in both animals and plants [1, 2]. According to the current model, most of the miRNA genes are transcribed by RNA polymerase II [3, 4], resulting in the production of the primary microRNA precursors (pri-miRNAs) with 3′ polyadenylated tails. In this regard, most of the pri-miRNAs could be detected by RNA sequencing (RNA-seq) designed for messenger RNA profiling. After transcription, the pri-miRNAs are subject to Dicing body-mediated two-step cropping for miRNA maturation. Specifically, a pri-miRNA is firstly processed into a pre-miRNA (precursor microRNA), and then into a short duplex consisting of miRNA-5p and miRNA-3p [1, 2].

As a result of the wide application of the sRNA high-throughput sequencing (sRNA-seq) technology, an explosion of miRNA discovery happened during the last ten years. At the same time, the increasing number of the miRBase registries [5] indicates that the current miRNA population is far from being saturated. For miRNA discovery, identification of the hairpin-structured precursors along with the featured miRNA-5p and miRNA-3p clusters became the essential criterion. However, many young or species-specific miRNA precursors are still under evolution [6], which leads to an obstacle for the researchers to distinguish them from the analogous stem-loop structures. Fortunately, tracking the miRNA processing intermediates could be helpful for miRNA annotation, since the processing signals were produced from Dicing body-mediated maturation. Degradome sequencing (degradome-seq) is a high-throughput sequencing technology for detecting the 3′ cleaved remnants of the transcripts with polyadenylated tails. It was widely used for mapping the miRNA-mediated target cleavage sites, especially in plants. Considering the fact that most of the miRNA genes were transcribed by RNA polymerase II, our group previously proposed the novel utility of degradome-seq data in tracking miRNA processing signals [7–10], which could facilitate miRNA discovery and validation. In this study, an integrated pipeline called PmiRDiscVali (https://github.com/unincrna/pmirdv) was developed for large-scale microRNA discovery in plants.

## Implementation

### PmiRDiscVali: A featured pipeline for large-scale miRNA prediction

To date, several powerful bioinformatics tools have been reported for large-scale discovery of animal miRNAs, such as miRDeep [11], miRDeep2 [12], miRDeep* [13], miRanalyzer [14], miRTRAP [15] and MIReNA [16]. They were developed according to the previously proposed criteria for miRNA annotation in animals [17]. Although the pathways of miRNA biogenesis and action are to some extent similar between animals and plants [1, 2], several distinct features should be taken into account when annotating the plant miRNAs [18]. For example, compared to those in animals, the plant miRNA precursors varies greatly in their length range. In this regard, specific parameters were introduced into the prediction tools for plant miRNAs, such as miRDeep-P [19] and miRPlant [20]. miRDeep-P, derived from miRDeep, is a computational tool popularly used for plant miRNA discovery. One of the advantages by using miRDeep-P for plant miRNA prediction is that the analysis does not depend on the availability of reference genomes [19]. However, miRDeep-P does not offer user-friendly prediction outputs, which may become an obstacle for researchers during result interpretation. Another tool, miRPlant, is a derivate of miRDeep*. It improved user experience through graphic presentation of the prediction results [20]. Unfortunately, the analysis by using miRPlant is highly dependent on the availability of genomic information, indicating the infeasibility of this tool for RNA-seq data-based miRNA prediction. Besides, the above two tools did not recognize the value of degradome-seq data in miRNA validation. In this consideration, miRNA Digger [21] was recently developed for genome-wide extraction of miRNA candidates by searching for the degradome-supported miRNA processing sites. However, it cannot be applied to the species without reference genomes, and no graphic output is available for the users. In this study, an integrated pipeline PmiRDiscVali (available at https://github.com/unincrna/pmirdv) was developed for large-scale identification of plant miRNAs. Based on RNA-seq and sRNA-seq data, the pipeline was designed to be compatible for both reference-based and de novo predictions. Referring to the plant miRNA registries in miRBase (release 21), sequence conservation analysis could be performed for the miRNA candidates by using PmiRDiscVali. If available, the degradome-seq data would be used to seek for the processing signals on the predicted miRNA precursors, thus enabling users to check the reliability of each candidate.

Taken together, PmiRDiscVali integrated miRDeep-P by considering its advantage in RNA-seq data-based prediction of plant miRNAs. On the other hand, PmiRDiscVali greatly improved the user experience through figure- and table-based result presentation. The user manual of PmiRDiscVali is available as the Additional file 1: Data S1 for the researchers.

### Data input and pre-treatment

**Fig. 1:**
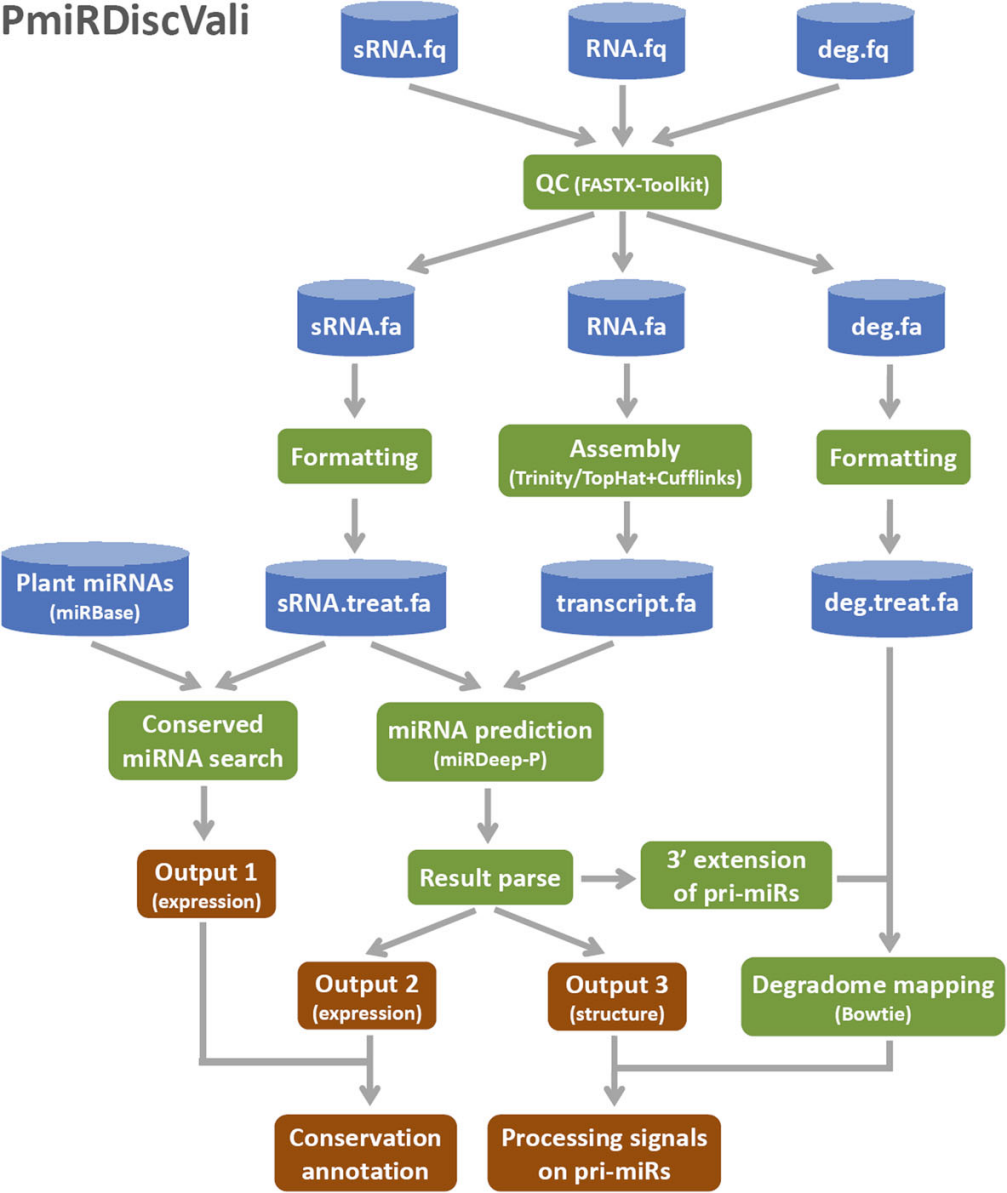
Flowchart diagram of the integrated pipeline PmiRDiscVali. The raw small RNA sequencing (sRNA-seq) and RNA sequencing (RNA-seq) data in FASTQ format was pre-treated and converted to FASTA format by FASTX-Toolkit. The sRNA-seq data was used to search for the conserved mature microRNA (miRNA) candidates according to the current plant miRNA registries in miRBase (release 21). Relying on the availability of the reference genome, distinct strategy (reference-based or de novo) was employed for transcriptome assembly by using the processed RNA-seq data. Then, the assembled transcripts and the sRNA-seq data were used for miRNA prediction by using miRDeep-P. The prediction results were parsed by an in-house Perl script, in order to graphically present the structures and the sRNA expression patterns of the miRNA precursors. If the degradome sequencing (degradome-seq) data was available, the pipeline would perform degradome signature mapping to seek for the processing evidence supporting the miRNA candidates

Starting from the raw sequencing data (including RNA-, sRNA- and degradome-seq data) in FASTQ format, the users are recommended to use FASTX-Toolkit (http://hannonlab.cshl.edu/fastx_toolkit/index.html) for data quality check and FASTA format conversion (Fig. 1). Then, the qualified sRNA- and degradome-seq data sets are normalized (in RPM, reads per million) and formatted by an in-house Perl script, thus ensuring them to be discernable by PmiRDiscVali. During the pre-treatment, the sequence length range of the sRNA reads (adjustable parameter) was set from 15 to 40 nt in default. For the degradome-seq reads longer than 20 nt, the 20-nt 5′ part of each read will be collected for further analysis. However, the degradome reads shorter than 20 nt will be discarded (the parameter “20 nt” is also adjustable). According to the genome availability of the species analyzed, the pre-treated RNA-seq data will be subject to de novo transcriptome assembly by using Trinity [22], or reference genome-based transcriptome assembly by using TopHat and Cufflinks [23, 24].

### Prediction and result output

The prediction workflow of PmiRDiscVali was divided into two parts. First, based on the plant miRNA registries in miRBase [release 21; this version includes a total of 8496 miRNA entries (4802 unique sequences) of 73 plant species] and sRNA-seq data, the conserved miRNA candidates along with their expression levels could be obtained. Second, the assembled transcriptome and the pre-treated sRNA-seq data are submitted to miRDeep-P for miRNA prediction. The prediction results are parsed by an in-house Perl script to provide a table summarizing miRNA expression levels and sequence conservation. The secondary structures of the miRNA precursors are predicted and drawn by using RNAplot included in Vienna RNA package 2.0 [25]. Besides, the sRNA accumulation pattern on each precursor is also graphically presented. If degradome-seq data is available, the pri-miRNA with 3′ 20-nt (adjustable parameter) extension retrieved from the assembled host transcript will be subject to degradome signature mapping by using Bowtie [26]. Four processing sites are defined on a pri-miRNA, i.e. the 5′ and 3′ ends of the 5′-armed mature miRNA, and the two ends of the 3′-armed miRNA. As a result, the degradome-supported processing sites will be identified on the pri-miRNAs, which could serve as one piece of strong evidence for the miRNA candidates.

## Results and discussion

### Case study

To confirm the utility of our pipeline, a case study was performed by using the sequencing data sets of *Dendrobium officinale*, which were reported by a recent study [27]. The high-throughput sequencing data used for this analysis includes: (1) The RNA-seq data of flowers (two replicates, NCBI SRA accession IDs: SRR2014396 and SRR2014476), leaves (two replicates: SRR2014297 and SRR2014325), roots (two replicates: SRR2014227 and SRR2014230) and stems (two replicates: SRR2014236 and SRR2014246); (2) The sRNA-seq data of flowers (two replicates: SRR2014148 and SRR2014149), leaves (two replicates: SRR2014146 and SRR2014147), roots (two replicates: SRR2014142 and SRR2014143) and stems (two replicates: SRR2014477 and SRR2014478); (3) The degradome-seq data of flowers (SRR2012592), leaves (SRR2012580), roots (SRR2012529) and stems (SRR2012531). The above three types of sequencing data were divided into four groups according to their organ origins (i.e. flowers, leaves, roots and stems), and were analyzed separately. For each organ, the two replicates of RNA-seq data were treated together for de novo transcriptome assembly.

Based on the list of the miRBase-registered plant mature miRNAs (release 21), a total of 240, 174, 135 and 154 unique sRNA sequences were identified from flowers, leaves, roots and stems respectively, which were regarded as the conserved miRNA candidates in *Dendrobium officinale* (Additional file 2: Table S1). Results of miRDeep-P prediction showed that 122 and 108 mature miRNA candidates were identified from flowers and leaves respectively, while only four and four candidates were discovered from roots and stems respectively. These miRNA candidates could be mapped onto 61, 54, two and two pri-miRNAs assembled from the RNA-seq data of flowers, leaves, roots and stems respectively. The processing of 26, 41, one and two pri-miRNAs was supported by the degradome-seq data of the above four organs, respectively. In this case, the degradome reads mapped to the four sites, i.e. the 5′ ends of the 5′- and the 3′-armed mature miRNAs, and 1-nt downstream of the 3′ ends of the two miRNAs, were considered to be the processing signatures. As a result, 39 sites on the 26 pri-miRNAs identified from flowers, 72 sites on the 41 pri-miRNAs from leaves, two sites on one pri-miRNA from roots, and three sites on the two pri-miRNAs from stems were regarded as degradome-supported processing sites (Additional file 2: Table S1).

**Fig. 2:**
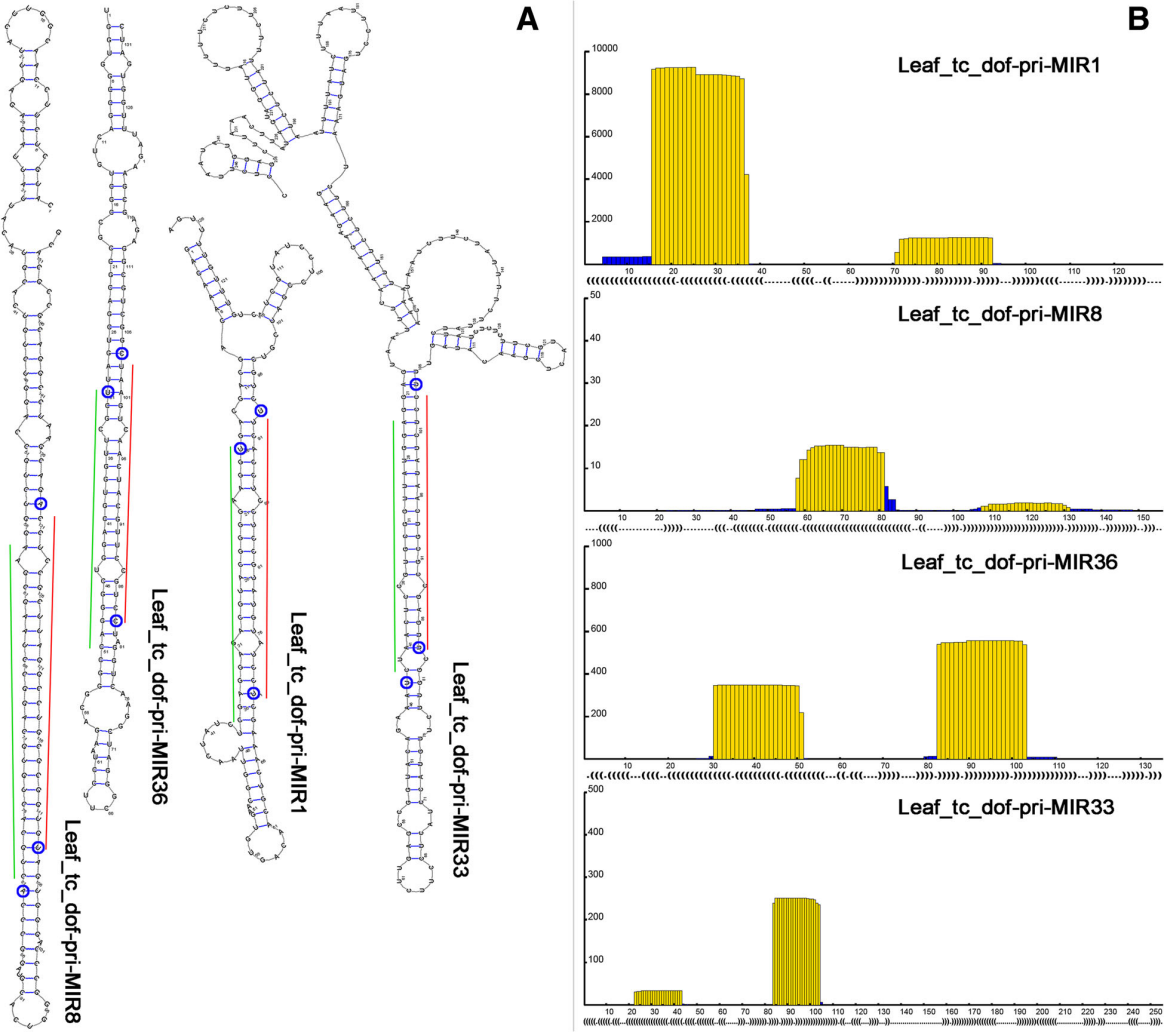
Exemplified graphic outputs of the case study by using PmiRDiscVali. **a** Secondary structures of the pri-miRNAs (primary microRNAs) predicted by using the sequencing data of the leaf organ of *Dendrobium officinale*. The mature miRNAs located on the 5′ arms of the pri-miRNAs were marked by green lines, and those located on the 3′ arms were marked by red lines. The processing sites supported by degradome sequencing data were highlighted by blue circles on the pri-miRNAs. **b** A global view of the small RNA (sRNA) expression levels on the predicted pri-miRNAs. The total counts of the sRNAs mapped onto the pri-miRNAs from different sRNA sequencing (sRNA-seq) data sets (four sequencing data sets, including flowers, leaves, roots and stems, in the case study) are shown together on the histograms. For each histogram, the *x* axis indicates the nucleotide position on each pri-miRNAs, and the regions predicted to encode the mature miRNAs were in yellow color. The *y* axis measures the total accumulation levels (in RPM, reads per million) of the sRNAs mapped onto each pri-miRNA. The dot-bracket notation below each histogram show the base pairing status of the secondary structure of the miRNA precursor. The bracket indicates a paired base, while the dot indicates an unpaired base

Fig. 2 provides an example of the four pri-miRNA candidates identified from the leaves of *Dendrobium officinale*. The graphic outputs of PmiRDiscVali show the stem-loop structures of the four pri-miRNAs, including Leaf_tc_dof-pri-MIR1, Leaf_tc_dof-pri-MIR8, Leaf_tc_dof-pri-MIR33 and Leaf_tc_dof-pri-MIR36 (Fig. 2a). On each precursor, a highly complementary region could be formed between the 5′- and the 3′-armed mature miRNAs. Three out of the four processing sites were supported by the degradome signatures, indicating the high reliability of the miRNA candidates. Based on the sRNA-seq data of the four organs, a global view of the sRNA expression pattern on each pri-miRNA was generated by PmiRDiscVali (Fig. 2b). On each pri-miRNA, the two mature miRNA-coding regions were covered by sharp expression peaks. Thus, it provided another piece of supporting evidence for the miRNA candidates. Besides, PmiRDiscVali also generated a table summarizing the organ-specific expression patterns of the predicted mature miRNAs (Table 1). Based on this table, we observed that both Leaf_tc_dof-miR1-5p and Leaf_tc_dof-miR1-3p were highly expressed in leaves, roots and stems compared to flowers. Sequence conservation analysis showed that Leaf_tc_dof-miR36 and Leaf_tc_dof-miR33 shared high sequence identity with the mature miRNAs of the miR168 and miR171 families respectively (Table 1). However, Leaf_tc_dof-miR8 was regarded as a novel miRNA which has not been registered in miRBase (release 21). Interestingly, both Leaf_tc_dof-miR1-5p and Leaf_tc_dof-miR1-3p shared similar but not identical sequences with the mature miRNAs of the miR528 family, indicating that their precursor might be under an evolutionary way towards the formal miR528 precursor.

**Table 1:** Example of the output table showing the expression values and sequence conservation of the mature microRNAs predicted in *Dendrobium officinale*

Mature ID	Mature sequence	Conservation	Mature expression (RPM)			
			Flower	Leaf	Root	Stem
Leaf_tc_dof-miR8-5p	AAGCGAAUCCGGACGUGUCACGUC	–	0.73	3.27	1.86	1.15
Leaf_tc_dof-miR8-3p	UGUGGCGCGUCCGGAUUCGCCUCC		0.00	0.13	0.00	0.00
Leaf_tc_dof-miR36-5p	UCGCUUGGUGCAGGUCGGGAC	Identical to miR168	222.74	107.83	70.50	132.34
Leaf_tc_dof-miR36-3p	CCUGCCUUGCAUCAACUGAAU		412.69	255.38	241.41	469.93
Leaf_tc_dof-miR1-5p	UGGAAGGGGCAUGCAGAGGAGC	Similar but not identical to miR528	183.19	2066.05	2511.72	3565.33
Leaf_tc_dof-miR1-3p	UCCUAUGUAUGCCUCCUCCACU		9.64	269.71	400.61	333.55
Leaf_tc_dof-miR33-5p	AGGUAUUGGCGUGCCUCAAUC	Identical to miR171	0.49	15.29	10.73	3.28
Leaf_tc_dof-miR33-3p	UUGAGCCGCGUCAAUAUCUCC		5.68	111.02	42.38	68.30

Summarily, the case study demonstrated the utility of PmiRDiscVali in transcriptome-wide identification of plant miRNA candidates. Some interesting conclusions could be inferred from the clear and concise result presentation. The results of this case study could be found at https://github.com/unincrna/pmirdv.

## Conclusions

Here, we reported an integrated pipeline PmiRDiscVali for transcriptome-wide prediction of plant miRNAs. Different from the previous tools that mainly focus on the importance of secondary structures and sRNA expression in miRNA prediction, PmiRDiscVali takes advantage of degradome-seq data to seek for processing signatures on the predicted precursors. Thus, this pipeline enables users to examine the reliability of the miRNA candidates from another angle. Besides, the graphic outputs of PmiRDiscVali including secondary structures, sRNA expression levels and processing signals, along with a summary table showing sequence conservation, improve user experience for result interpretation. Notably, by replacing miRDeep-P with miRDeep, PmiRDiscVali could be modified as a computational pipeline for miRNA prediction in animals. Finally, we hope that PmiRDiscVali will become a popular miRNA prediction tool for the plant biologists. It could provide the users with the focused lists of relatively reliable miRNA candidates.

## Availability and requirements

Project name: PmiRDiscVali

Project home page: https://github.com/unincrna/pmirdv

Operating system(s): Linux/Unix software environment

Programming language: Perl

Other information is available in the user manual (Additional file 1: Data S1).

## Additional files

Additional file 1: **Data S1.** User manual of PmiRDiscVali. (PDF 259 kb)

Additional file 2: **Table S1.** Result summary of the case study on the miRNA prediction in *Dendrobium officinale*. (PDF 88 kb)

## Abbreviations

degradome-seq: Degradome sequencing
miRNA: microRNA
pri-miRNA: Primary microRNA
RNA-seq: RNA sequencing
RPM: Reads per million
sRNA: Small RNA

## References

[1] Bartel DP (2004). MicroRNAs: genomics, biogenesis, mechanism, and function. Cell.

[2] Jones-Rhoades MW, Bartel DP, Bartel B (2006). MicroRNAS and their regulatory roles in plants. Annu Rev Plant Biol.

[3] Lee Y, Kim M, Han J, Yeom KH, Lee S, Baek SH, Kim VN (2004). MicroRNA genes are transcribed by RNA polymerase II. EMBO J.

[4] Xie Z, Allen E, Fahlgren N, Calamar A, Givan SA, Carrington JC (2005). Expression of Arabidopsis MIRNA genes. Plant Physiol.

[5] Kozomara A, Griffiths-Jones S (2014). miRBase: annotating high confidence microRNAs using deep sequencing data. Nucleic Acids Res.

[6] Berezikov E (2011). Evolution of microRNA diversity and regulation in animals. Nat Rev Genet.

[7] Ma X, Tang Z, Qin J, Meng Y (2015). The use of high-throughput sequencing methods for plant microRNA research. RNA Biol.

[8] Meng Y, Gou L, Chen D, Wu P, Chen M (2010). High-throughput degradome sequencing can be used to gain insights into microRNA precursor metabolism. J Exp Bot.

[9] Yu D, Ma X, Zuo Z, Shao W, Wang H, Meng Y (2017). Bioinformatics resources for deciphering the biogenesis and action pathways of plant small RNAs. Rice.

[10] Yu D, Xu M, Ito H, Shao W, Ma X, Wang H, Meng Y (2018). Tracking microRNA processing signals by Degradome sequencing data analysis. Front Genet.

[11] Friedlander MR, Chen W, Adamidi C, Maaskola J, Einspanier R, Knespel S, Rajewsky N (2008). Discovering microRNAs from deep sequencing data using miRDeep. Nat Biotechnol.

[12] Friedlander MR, Mackowiak SD, Li N, Chen W, Rajewsky N (2012). miRDeep2 accurately identifies known and hundreds of novel microRNA genes in seven animal clades. Nucleic Acids Res.

[13] An J, Lai J, Lehman ML, Nelson CC (2013). miRDeep*: an integrated application tool for miRNA identification from RNA sequencing data. Nucleic Acids Res.

[14] Hackenberg M, Sturm M, Langenberger D, Falcon-Perez JM (2009). Aransay AM: miRanalyzer: a microRNA detection and analysis tool for next-generation sequencing experiments. Nucleic Acids Res.

[15] Hendrix D, Levine M, Shi W (2010). miRTRAP, a computational method for the systematic identification of miRNAs from high throughput sequencing data. Genome Biol.

[16] Mathelier A, Carbone A (2010). MIReNA: finding microRNAs with high accuracy and no learning at genome scale and from deep sequencing data. Bioinformatics.

[17] Ambros V, Bartel B, Bartel DP, Burge CB, Carrington JC, Chen X, Dreyfuss G, Eddy SR, Griffiths-Jones S, Marshall M (2003). A uniform system for microRNA annotation. RNA.

[18] Meyers BC, Axtell MJ, Bartel B, Bartel DP, Baulcombe D, Bowman JL, Cao X, Carrington JC, Chen X, Green PJ (2008). Criteria for annotation of plant MicroRNAs. Plant Cell.

[19] Yang X, Li L (2011). miRDeep-P: a computational tool for analyzing the microRNA transcriptome in plants. Bioinformatics.

[20] An J, Lai J, Sajjanhar A, Lehman ML, Nelson CC (2014). miRPlant: an integrated tool for identification of plant miRNA from RNA sequencing data. BMC Bioinformatics.

[21] Yu L, Shao C, Ye X, Meng Y, Zhou Y, Chen M (2016). miRNA digger: a comprehensive pipeline for genome-wide novel miRNA mining. Sci Rep.

[22] Grabherr MG, Haas BJ, Yassour M, Levin JZ, Thompson DA, Amit I, Adiconis X, Fan L, Raychowdhury R, Zeng Q (2011). Full-length transcriptome assembly from RNA-Seq data without a reference genome. Nat Biotechnol.

[23] Ghosh S, Chan CK (2016). Analysis of RNA-Seq data using TopHat and cufflinks. Methods Mol Biol.

[24] Trapnell C, Roberts A, Goff L, Pertea G, Kim D, Kelley DR, Pimentel H, Salzberg SL, Rinn JL, Pachter L (2012). Differential gene and transcript expression analysis of RNA-seq experiments with TopHat and cufflinks. Nat Protoc.

[25] Lorenz R, Bernhart SH, Honer Zu Siederdissen C, Tafer H, Flamm C, Stadler PF, Hofacker IL (2011). ViennaRNA Package 2.0. Algorithms Mol Biol.

[26] Langmead B, Trapnell C, Pop M, Salzberg SL (2009). Ultrafast and memory-efficient alignment of short DNA sequences to the human genome. Genome Biol.

[27] Meng Y, Yu D, Xue J, Lu J, Feng S, Shen C, Wang H (2016). A transcriptome-wide, organ-specific regulatory map of Dendrobium officinale, an important traditional Chinese orchid herb. Sci Rep.

