# Supplementary material for: PmiRDiscVali: an integrated pipeline for plant microRNA discovery and validation
Source: BMC Genomics. 2019 Feb 13;20:133. doi: 10.1186/s12864-019-5478-7 (PMC6375137; doi:10.1186/s12864-019-5478-7)
Supplement: Supplementary file 1 — Data S1. User manual of PmiRDiscVali. (PDF 259 kb) [file 12864_2019_5478_MOESM1_ESM.pdf]

User Manual

1. INTRODUCTION **2**
2. REQUIREMENT **3**
2.1 REQUIRED 3
(1) PERL PACKAGES 3
(2) BOWTIE 3
(3) MIRDEEP-P 4
(4) VIENNA RNA PACKAGE 4
2.2. OPTIONAL 6
(1) FASTX-TOOLKIT 6
(2) TRINITY 6
(3) TOPHAT + CUFFLINKS 7
3. PREPARATION OF INPUT FILES **8**
3.1 TRANSCRIPTOME 8
3.2 SRNA-SEQ DATA 8
3.3 DEGRADOME-SEQ DATA 8
3.4 JOBLIST 9
3.5 KNOWN MIRNAS 10
4. *PMIRDISCVALI* PACKAGE **10**
4.1 QUICKSTART 10
4.2 DESCRIPTION OF THE SCRIPTS 11
5. OUTPUT FILES **16**
5.1 FILES 16
5.2 DIRECTORIES 17
REFERENCES: **18**

1. Introduction

MicroRNAs (miRNAs) play important regulatory roles in diverse biological processes in plants and animals. Identification of miRNAs is always the startingpoint to understand their biological roles and many bioinformatics tools have beenavailablecurrently for this purpose. In addition, to enhance the prediction reliability, theprediction tools mainly focus on the analyses of secondary structures and small RNA (sRNA) expressionpatterns of the miRNA precursors.

Thepresent pipeline,*PmiRDiscVali*,is developed forplant miRNA discoverythat identifies the miRNAs by both homology search and *de novo* prediction (by integrating miRDeep-P). Notably, besides the structures of primary miRNAs and expression of the mature miRNAs, this pipeline also investigates the processing signals from the miRNA precursors by using the degradome-seq data,which therefore allowing users to check the prediction reliability from another angle.

NOTE:

The complete *PmiRDiscVali* pipeline works based on the qualified data from sRNA-seq, degradome-seq and the constructed transcriptome, but it would also work when the degradome-seq datalacks, for which the only difference is the unavailability of processing signalsin the last result.

Contact information:

Dongliang Yu (yudl@hznu.edu.cn)

YijunMeng (mengyijun@zju.edu.cn)

2. Requirement

PmiRDiscVali pipeline contains a package of perl scripts and requires the Linux/Unix software environment. In addition, the followingthird-party tools are also needed before starting.

2.1 Required

(1) Perl packages

Standard Perl should be available.

The **SVG**perl package is required for graphing, which can be installed via the Perl CPAN module, *i.e.*,

```
perl -MCPAN -e shell
install SVG
```

or can be downloaded from CPAN (*search.cpan.org*) andinstalled in the hard way according to its instruction.

The **Bioperl** package is also required for sequence feature statistics, which can be installed via the Perl CPAN module, *e.g.*,

```
perl -MCPAN -e shell
d /bioperl/
install C/CJ/CJFIELDS/BioPerl-1.007001.tar.gz
```

Or via the other method provided by https://bioperl.org/INSTALL.html.

(2) bowtie

bowtie(version 1.1.2) is used in this pipeline to align the sRNA-seq data and degradome-seq data to the pri-miRNAs(1).To install bowtie, download thelatest binary pack from *sourceforge.net/projects/bowtie-bio/files/bowtie/*,unpack the zip archive and

copy the bowtie executables to a directory in your PATH, *e.g.*,

```
unzip bowtie-1.1.2-linux-x86_64.zip
cd bowtie-1.1.2-linux-x86_64
cp bowtie $HOME/bin
cp bowtie-build $HOME/bin
```

Please check the bowtie manual for a more detailed description.

(3) miRDeep-P

miRDeep-P (v1.3),a package of perl scripts, is used for *de novo* prediction of miRNAs(2).Itcould be downloaded from *sourceforge.net/projects/mirdp/* and usually just need to be unpacked. The users could then copy these perl scripts to a directory in your PATH, *e.g.*,

```
tar zxvfmiRDP1.3.tar.gz
cd miRDP1.3
cp *.pl $HOME/bin
```

(4) Vienna RNA package

Vienna RNA package (v2.4.10)could be downloaded from www.tbi.univie.ac.at/RNA/#download(3).

To install it, the users need to unpack the compressed file, configure and make, as well as add the src/bin to the user's PATH, *e.g.*,

```
tar -zxvf ViennaRNA-2.4.10.tar.gz
cd ViennaRNA-2.4.10
./configure
make
make install
export PATH=$PATH:/home/bio/ViennaRNA-2.3.5/src/bin
```

The function **RNAfold** is required by**miRDeep-P** and the function**RNAplot**is required in displaying the pri-miRNA structures and marking the degradome-supported processing signals.

2.2. optional

We assume the users have acquired the qualified transcriptome andprocessed data from sRNA-seq and degradome-seqwhen starting the *PmiRDiscVali* pipeline. But, if the users only have raw data from sequencing platforms, the following tools may then be help for preparing the input data.

(1)FASTX-Toolkit

FASTX-Toolkit was used when testing the pipeline to remove the adaptors and filter low-quality reads(*e.g.*, with the number of Q30 base fewer than 50% of the read length). For example:

To remove the adaptor:

```
fastx_clipper -a TGGAATTCTCGGGTGCCAAGG -Q 33 -iin.fq -o out1.fq
```

To remove the low-quality reads:

```
fastq_quality_filter -q 30 -p 50 -Q 33 -i out1.fq -o out2.fq
```

To convert the FASTQ format to FASTA

```
fastq_to_fasta–Q 33 –i out2.fq –o out2.fa
```

The software and a more detailed description about its installation and usage could be retrieved from *http://hannonlab.cshl.edu/fastx_toolkit/index.html*.

(2) Trinity

Trinitycould be used for *de novo* assembly to generate the transcriptome for reference genome unavailable organisms(4). It could be downloaded from *https://github.com/trinityrnaseq/trinityrnaseq/releases*and installed with 'make', *e.g.*,

```
tar zxvf Trinity-v2.8.4.tar.gz
cd Trinity-v2.8.4
make
```

A simple example of trinity assembly (for pair-end library):

```
Trinity --seqTypefq --JM 50G --left data1_1.fq --right data1_2.fq --CPU 20
```

(3)TopHat+Cufflinks

TopHat (http://ccb.jhu.edu/software/tophat/index.shtml) and Cufflinks (http://cole-trapnell-lab.github.io/cufflinks/install/) could be used for reference genome-guided assembly(5).To install TopHat, download the latest version (2.1.1) of binary package, unpack and copy the executable files to a directory in your PATH, *e.g.*,

```
tar zxvftophat-2.1.1.Linux_x86_64.tar.gz
cd tophat-2.1.1.Linux_x86_64
cp * $HOME/bin
```

To install Cufflinks, download the binary tarball (2.2.1), unpackand copy the executable files to a directory in your PATH, *e.g.*,

```
tar zxvfcufflinks-2.2.1.Linux_x86_64.tar.gz
cd cufflinks-2.2.1.Linux_x86_64
cp * $HOME/bin
```

A simple example of reference genome guided transcriptome assembly by using TopHat+cufflinks:

```
tophat-p 10 -G OA.gtf -o map_outgenome rep1_1.fq rep1_2.fq
cufflinks -p 10 -o trans_outmap_out/accepted_hits.bam
```

3. Preparation of input files

3.1transcriptome

FASTA files in a directory like ‘cDNA_dir’.

3.2sRNA-seq data

Normalized sRNA-seq datain FASTA format, deposited in a directory like ‘sRNA_dir’.The normalized sRNA-seqfiles in FASTA format areorganized as:

```
>S3456_54632_18.3_x1342
AGGCTACTACCGCTA
```

Specifically, "S3456": sRNA-seqdataset ID; "54632": unique ID for the sRNA sequence; "18.3": the normalized count (in RPM, reads per million) of the sRNA; "1342": the raw count of the sRNA.

In the case of raw sRNA-seq data (FASTQ format), the users could use FASTX-Toolkit to get qualified FASTAfiles. Then, the FASTA files could be normalized by using the script**format_sRNA.pl.**

e.g.,

```
perl format_sRNA.pl in_dirsRNA_dir 15 40
```

We assume the qualified sRNA FASTA files are stored in "in_dir" and the normalized files would be stored in "out_dir". sRNAs with the length ranged from ‘15’ntto‘40’nt are collected for further analyses.Users can adjust the size of selected sRNAsaccording to their own understanding.

3.3degradome-seq data

Normalized degradome-seq data in FASTA format, deposited in a directory like ‘deg_dir’.For the raw data (FASTQ), please use FASTX-Toolkit to get qualified FASTA files. Then, use the script**format_degradome.pl**to extract the first 20 nt

at the 5’ ends of the degradome sequences and perform the normalization, *e.g.*,

```
perl format_degradome.pl in_dirdeg_dir 20
```

We assume the qualified FASTA files from degradome-seq are stored in "in_dir" and the normalized files would be stored in "deg_dir".The length to extract from the 5' ends is specified by "20" (optional, users can change length according to their own understanding).

The formatted filesare organized as follows:

```
>S246721_36732_4.5
TACTACAGGCTACTACCGCT
```

Specifically,"S246721": degradome-seq data set ID; "36732": unique ID for thedegradome sequence; "4.5": the normalized count (in RPM) of the degradome sequence.

3.4joblist

A plain textfile**.**The‘joblist’ should be organized as:

```
<transcriptome_file><sRNA-seq_data><degradome-seq_data>
e.g.,
At.cdna.fna  srna_rep1/srna_rep2/srna_rep3   deg_rep1/deg_rep2
```

‘<>’ indicates the file names. Each line in the ‘joblist’ indicates a single task, which would be analyzed one by one by using *PmiRDiscVali*scripts.

If degradome-seq data is not provided, the last field should be marked as **‘NULL’**. If multiple data sets are available for sRNA- or degradome-seq data, ***‘/’*** should be used to separate the different data sets.

Please notice that each line in the file‘joblist’ should not start with a space, blank line is not allowedeither.

3.5known miRNAs

A plain text file, assigned by parameter '--ref' of PmirDV.pl.This is a list of known (conserved) miRNAs from miRBase, organized as <temporary ID><unique miRNA sequence><miRNA identifiers in miRBase>, like

```
plantmiR1AGGGGGCAATCTCACCTCAACata-miR9772b-3p&ata-miR9772a-3p
```

It could be substituted by any other miRNAs the users interested in that are organized with the same format. By default, it is the provided 'plantmiR.list'.

4. *PmiRDiscVali* package

4.1 Quickstart

The *PmiRDiscVali*pipelinecan be started by*PmirDV.pl* by oneline command, *e.g.*,

```
perlPmirDV.pl --org dof--joblisttest.list --cdnacDNA_dir --srnasRNA_dir
--degdeg_dir

parameters:
--help|-h : Usage
--org          : Organism(abbreviation,like ath);
--joblist: Jobinformation,eachtask per line [String];
--proc|-p : Number of processors to use [Integer,default 3];
--exlen: Length of pri-miRNA extension [Integer,default 50];
--cdna        : Directory contains transcriptome files [Default: ./];
--srna        : Directory contains sRNA files [Default: ./];
--deg        : Directory contains degradome files [Default: ./];
--ref          : known miRNAs [Default: ./plantmiR.list];
```

The tasks are described in the 'test.list' (**Section 'Preparation of input files'**). The transcriptome, sRNA-seq and degradome-seq data are respectively stored in the directories designated by parameters '--cdna', '--srna' and

'--deg'.Result of each task would be generated and delivered into the directory named by the prefix of transcriptome file(Details in**Section 'Output files'**).

4.2 Description of the scripts

(1)get_contig_len.pl

Before miRNA prediction, lengths of transcripts involved in current taskare calculated by this script,which works as

```
perl get_contig_len.pl <cdna_dir><transcriptome>

parameters:
cdna_dir        : directory includes the transcriptome files;
transcriptome   : file, transcriptome involved in current task;
```

that generates a file named *.len in the <cdna_dir>.

(2)step1_miRDP.pl

This script starts the *de novo* prediction of miRNAs.

```
perl step1_miRDP.pl <proc><cdna_dir><transcriptome><sRNA_dir>
<sRNA_file_list>

parameters:
proc            :Number of processors to launch;
cdna_dir        :Directory includes the transcriptome files;
transcriptome   :File, transcriptome involved in current task;
sRNA_dir        :Directory includes the normalized sRNA files;
sRNA_file_list  :Names of sRNA files split by '/';
```

Once input the transcripts and sRNAs, standard miRDeep-P is used then to predict the miRNAs. As recommended, up to '250'nt is selected when excisingcandidatepri-miRNAs in the examples, which could be adjusted in line 43 of this script.

(3)step2_parse_miRDP.pl&step3_miRDP_mature_star_parse.pl

These two scripts parse the result from miRDeep-P and save it as a table (*.sort), which contains the positions and sequences of predicted miRNAs, as well as the sequences and structures of predicted pre- and pri-miRNAs.

```
perl step2_parse_miRDP.pl <transcriptome><sRNA_file_list>
perlstep3_miRDP_mature_star_parse.pl <known_miRNAs><transcriptome>

parameters:
transcriptome      :File, transcriptome involved in current task;
sRNA_file_list     :Names of sRNA files split by '/';
known_miRNAs       :Plain text file contains known miRNAs;
```

(4) step4_add_ex.pl

This script is used to extend the pri-miRNAs at the 3 prime end with the length assigned by the <extension length> (parameter 'exlen' of PmirDV.pl).It generates files '*.addex' that add the information of'Extended_pri-seq' to the results from step3.

```
perl step4_add_ex.pl <extension length><transcriptome><cdna_dir>

parameters:
extension length   :extend the predicted pri-miRNAs with a length up to this value;
transcriptome      :File, transcriptome involved in current task;
cdna_dir           :Directory includes the transcriptome files;
```

(5)step5_GetExPriAndMap.pl

This step maps the degradome-seq data to the extended pri-miRNAs using bowtie to seek fordegradome-based cropping evidence. It generates the files

'*.degsignal' that add an column of 'degradation_sigal' to the results from step4.

perl step5_GetExPriAndMap.pl <proc><transcriptome><deg_dir><deg_list>

parameters:

proc:Number of processors to launch;

transcriptome:File, transcriptome involved in current task;

deg_dir:Directory includes the normalized degradome files;

deg_list:Names of degradome files split by '/';

(6) step6_Name.pl&**step7_Suminfor.pl**

These two scripts are used to generate the sequence files of pre- and pri-miRNAs (see *.pre-miRNA.seq& *.pri-miRNA.seq), as well as a summary file of the miRNAspredicted by miRDeep-P (see *-prediction.xls). Scripts are run by the commands:

perl step6_Name.pl <organism><transcriptome>

perl step7_Suminfor.pl <organism><transcriptome>

parameters:

organism :*Abbreviation of the organism;*

transcriptome :File, transcriptome involved in current task;

Notably, step4 to step7 are required for tasks with degradome-seq data. If degradome-seq data is not available, instead of the above four steps (**step4**to **step7**),only **step6_Name_NODEG.pl** and **step7_Suminfor_NODEG.pl** are used to generate the pre- and primiRNAs and a summary information of miRDeep-P prediction.

(7)step8_GetExpressionProfile.pl

This script is used to extract the expression values (RPM) of miRDeep-P-predicted miRNAs from the sRNA-seq data sets, *e.g.*,

```
perl step8_GetExpressionProfile.pl
<organism><transcriptome><sRNA_dir><sRNA_file_list>

parameters:
organism        : Abbreviation of the organism;
transcriptome   :File, transcriptome involved in current task;
sRNA_dir        :Directory includes the normalized sRNA files;
sRNA_file_list  :Names of sRNA files split by '/';
```

For the details of the output file (*-miRNA_expression.xls), see **Section 'Output files'**.

(8) step9_Plot_expression.pl

Thisis used to align the sRNA-seq data to the pri-miRNAs andcalculate the coverage of everyposition. The result are showed using both text files and figures (see **priseq_exp_fig&priseq_exp_table**).

(directory priseq_exp_table) and graphs (directory priseq_exp_fig).

```
perl step9_Plot_expression.pl
<organism><transcriptome><result_dir><srna_dir><srna_list><proc>
parameters:
organism        :Abbreviation of the organism;
transcriptome :File, transcriptome involved in current task;
sRNA_dir        :Directory includes the normalized sRNA files;
sRNA_file_list  :Names of sRNA files split by '/';
proc            :Number of processors to launch;
```

(9) step10_plot_structures.pl

This script is used to plot the pri-miRNAstructures, with mature miRNAs at 5

and 3 primes colored green and red, respectively, as well as the processing signal marked by a circle, *e.g.*,

```
perl step10_plot_structures.pl <org><transcriptome>
parameters:
org              : Abbreviation of the organism;
transcriptome :File, transcriptome involved in current task;
```

(10) conserve_miRNA_search.pl

This script is used to identify the conservedmiRNAs as well as their abundance from the given sRNA-seq data. The result is save in the file '*_conserved_miR.xls'.

```
perl conserve_miRNA_search.pl
<organism><known_miRNAs><transcriptome><sRNA_dir><srna_list>
parameters:
organism         : Abbreviation of the organism;
known_miRNAs     : Plain text file contains known miRNAs;
transcriptome :File, transcriptome involved in current task;
sRNA_dir         :Directory includes the normalized sRNA files;
sRNA_file_list   :Names of sRNA files split by '/';
```

(11) get_report.pl

This scriptis used to get the final report (*_summary.txt) (see **Section Output files**).

perlget_report.pl<organism><transcriptome><srna_list><degradome_list>

parameters:

organism : Abbreviation of the organism;

transcriptome :File, transcriptome involved in current task;

sRNA_list:Names of sRNA files split by '/';

degradome_list:Names of degradome files split by '/';

(12) clean.pl

perlget_report.pl<transcriptome>

parameters:

transcriptome :File, transcriptome involved in current task;

5. Output files

With the default settings, all of the output files are deposited in the "**result**" directory, including six files and three directories.For more detailed information of output files, please check the provided directory '**examples**'.

5.1Files

***summary.txt**: including the input data information of the current job and the statistics of the prediction results.

***conserved_miR.list**: a list of conserved miRNA candidates based on homology search, including mature miRNA sequences, miRBaseIDs and the expression values (RPM).

***miRNA_expression.xls**: the expression levels (RPM)of the mature

miRNAspredicted by miRDeep-P.

***prediction.xls**: a summary table showing the information of the miRNAspredicted by miRDeep-P, including: matuemiRNA IDs, the positions of the mature miRNAs on the pri-miRNAs, the homologous miRNAs inmiRBase, the IDs of pri-miRNAs/ pre-miRNAs, and the identified the degradomesupported cropping sites (a total of four sites, *i.e.*, 5' of mature miRNA-5p, 3'+1 nt of mature miRNA-5p, 5' of mature miRNA-3p, and 3'+1 nt of mature miRNA-3p. “1” stands for the sites supported by degradome signatures, while “0” stands for the sites without degradome evidence).

***pri-miRNA.seq/*pre-miRNA.seq**:sequence files of the pri-miRNAs and pre-miRNAs predicted by miRDeep-P.

5.2 Directories

priseq_exp_fig: histograms showing the accumulation levels (RPM) of the sRNAs on thepri-miRNAs with 1-nt resolution. For each job, the sRNA accumulation levels derive from the sum of all sRNA-seq data sets contained in this job.

priseq_exp_table: expression data used for drawing histograms.

structures: graphic presentation of the pri-miRNA structures predicted by miRDeep-P. Green color indicates the 5'-armed mature miRNAs, and red color indicates the 3'-armed mature miRNAs. If any, the degradome-supported cropping sites will be marked by circles.

References:

1. Langmead B, Trapnell C, Pop M, Salzberg SL. Ultrafast and memory-efficient alignment of short DNA sequences to the human genome. Genome Biology. 2009;10(3):R25.
2. Yang X, Li L. miRDeep-P: a computational tool for analyzing the microRNA transcriptome in plants. Bioinformatics. 2011;27(18):2614-5.
3. Lorenz R, Bernhart SH, Honer Zu Siederdissen C, Tafer H, Flamm C, Stadler PF, et al. ViennaRNA Package 2.0. Algorithms for molecular biology : AMB. 2011;6:26.
4. Haas BJ, Papanicolaou A, Yassour M, Grabherr M, Blood PD, Bowden J, et al. De novo transcript sequence reconstruction from RNA-seq using the Trinity platform for reference generation and analysis. Nature protocols. 2013;8(8):1494-512.
5. Trapnell C, Roberts A, Goff L, Pertea G, Kim D, Kelley DR, et al. Differential gene and transcript expression analysis of RNA-seq experiments with TopHat and Cufflinks. Nature protocols. 2012;7(3):562-78.
