# Supplementary material for: PmiRDiscVali: an integrated pipeline for plant microRNA discovery and validation
Source: BMC Genomics. 2019 Feb 13;20:133. doi: 10.1186/s12864-019-5478-7 (PMC6375137; doi:10.1186/s12864-019-5478-7)
Supplement: Supplementary file 2 — Table S1. Result summary of the case study on the miRNA prediction in Dendrobium officinale. (PDF 88 kb) [file 12864_2019_5478_MOESM2_ESM.pdf]

Table S1 Result summary of the case study on the miRNA prediction in *Dendrobium officinale*.

Organ	No. of the conserved mature miRNAs identified from miRBase registries (release 21)	miRDeep-P prediction results							
		No. of mature miRNAs (unique sequences)	No. of conserved mature miRNAs (unique sequences)	No. of pri-miRNAs	No. of degradome-supported pri-miRNAs	No. of processing sites supported by degradome-seq data			
						Site 1	Site 2	Site 3	Site 4
**Flower**	**240**	**122 (82)**	**26 (10)**	**61**	**26**	**17**	**0**	**8**	**14**
**Leaf**	**174**	**108 (92)**	**18 (11)**	**54**	**41**	**30**	**3**	**23**	**16**
**Root**	**135**	**4 (4)**	**1 (1)**	**2**	**1**	**0**	**1**	**1**	**0**
**Stem**	**154**	**4 (4)**	**0 (0)**	**2**	**2**	**1**	**0**	**2**	**0**

Note: "Site 1": The 5' end of the 5'-armed mature miRNA. "Site 2": The 3' end of the 5'-armed mature miRNA. "Site 3": The 5' end of the 3'-armed mature miRNA. "Site 4": The 3' end of the 3'-armed mature miRNA.
